# Limitations of Computational Morality: Can We Morally Enhance Ourselves via AI?

**DOI:** 10.1007/s11948-026-00634-8

**Published:** 2026-10-22

**Authors:** Florian Richter

**Affiliations:** Catholic University of Eichstätt-Ingolstadt, School of Transformation and Sustainability, Ostenstr. 26, 85072 Eichstätt, Germany

**Keywords:** Connectionism, Rule-based approaches, Computational ethics, Artificial intelligence

## Abstract

Computational or machine ethics examines how morality could be implemented in machines and represented computationally. In the cognitive sciences, different architectures (rule-based and connectionist) have been proposed. Nevertheless, such approaches have limitations when incorporating ethical claims. With the rise of artificial intelligence, new ways of simulating moral expertise seem possible, where chatbots can serve as dialogue partners for moral orientation. However, as I will argue, such systems lack a theoretical grounding in metaethics, as do the former approaches. In order to align such systems ethically and ground them conceptually, a topography of moral competence must be developed. By outlining this topography, I will show some directions for further research and how such systems might enhance us morally.

## Introduction

In 1993, Goldman asked whether moral concepts like good, fair, and just are “wholly determined by the cultural environment” or “innate structures” (Goldman, 1993, p. 623). The question needs to be reconsidered in light of the rise of neuroscience and artificial intelligence (AI). Are moral concepts (or our moral thinking) wholly determined by neural, biological, or psychological factors that can be examined empirically, or are there innate logical structures that determine our (moral) thinking? And could they be computationally elaborated for implementation on machines? This dualism also pervades the ethical discussion of the cognitive sciences and of how morality is modeled, even though some approaches mix methods. For that purpose, the cognitive architectures must be reconstructed to scrutinize the ethical foundations of this dualistic opposition.

It is a rather crude distinction to sort philosophy of mind into rationalistic and empiricist approaches. However, it seems that this kind of dualism has shaped debates since the time of Descartes and Hume. Yet in cognitive science, rule- or grammar-based approaches (Chomsky, 2015; Fodor, 2008) could be classified as rationalist, while connectionist, neuroscientific, and Bayesian approaches could be classified as empiricist (Thagard, 2012). It is unrealistic to assume that the mind can be described either as a purely biological or naturalistic phenomenon or as a computational, rule-based model. Both approaches fail to describe the mind adequately; instead, unifying approaches have been proposed, such as neuro-symbolic (Latapie et al., 2022) or embodied, grounded cognition (Barsalou, 2008). Furthermore, the human mind has been described as based on normative-linguistic (Brandom, 1994) or cooperative practices (Tomasello, 1999). Nevertheless, the ethical aspects and implications of the approaches have not been sufficiently scrutinized. Although experimental ethics and moral psychology have investigated ethical issues regarding cognition, the limitations of computational morality have not been thoroughly examined.

If the mind is merely a biological phenomenon, enhancing intelligent behavior through biological means, e.g., medicine, should also be morally permissible, as studies on the possibilities of neuroenhancement claim (Balconi & Angioletti, 2021; Dietz et al., 2016; Dündar-Coecke, 2021). However, such neuroenhancement approaches have mainly increased attention span or concentration, not other, more essential aspects of intelligence. In contrast, if the mind is a rule-governed phenomenon, enhancing intelligent behavior or the mind by technological means, e.g., brain-computer interfaces, should be morally permissible. Nonetheless, such applications are mostly therapeutic (Shih et al., 2012). Such issues presuppose a certain conception of “cognition” or the “mind.” Cognitive architectures are representations of human cognition that capture tasks such as memorizing, learning, and problem-solving. The problem is that ethical questions are treated as problem-solving tasks, in which rule-based approaches could propose standards and apply if … then rules to moral problems, or in which connectionist approaches could emulate problem-solving based on biological mechanisms. Both approaches have implications for the computational elaboration of morality, based on a crucial misunderstanding, and will be discussed in sections “Rule-Based Cognition” and “Connectionist Cognition”.

If technology is understood as a means to optimize performance, processes, or decisions, conflicts of values arise. For instance, enhancing memory or attention through pharmacological methods creates a value conflict between health and profitability/functionality. Students might be more attentive, but it can have repercussions for their health. A problematic aspect might be that the educational system is structured to reward the mindless absorption of knowledge, which can be enhanced by neurostimulants. Nevertheless, an educational system can also be designed so that one can still fail within it, even by taking pharmacological enhancements. A student who takes drugs to enhance memory and attention can fail in tests, since the tests might not only measure knowledge absorption but also problem-solving skills.

Emerging technologies, such as AI, can also be conditioning factors for shaping our practices, daily and professional lives: from smartphone applications to automated driving to chatbots based on large language models (LLMs) for tasks at work or in education. Such chatbots can also be used as so-called “artificial moral experts” (Cervantes et al., 2020; Lara & Deckers, 2020) to seek moral orientation, and users often do so but mostly quietly (Landes et al., 2026). This is concerning since these systems were not developed for that purpose, are even perceived as more trustworthy than human ethics experts (Dillion et al., 2025), and can subconsciously influence moral judgment (Krügel et al., 2023). This is why worries regarding manipulation and interference with our autonomous moral judgment are justified. *However, can they also enhance us morally?* Can they serve as helpful tools or systems for moral decision-making and orientation? These questions engage in debates in AI ethics about enhancement, nudges, manipulation, responsibility gaps, and value alignment of AI-based systems. A deeper analysis can also lead to reconceptualizing core ethical concepts, such as autonomy, moral judgment, and moral agency, in light of AI. To do so, AI-based moral enhancement must be systematically examined at the conceptual level and complemented by earlier approaches in computational morality, which more clearly elaborate on the underlying cognitive architectures.

Recently, moral enhancement via biomedical means has been widely debated (Persson & Savulescu, 2008; Savulescu, 2001; Paulo & Bublitz, 2019; Bostrom & Ord, 2006). Some of the debate’s metaethical shortcomings have also been reconstructed (Paulo, 2018). Like this debate, moral enhancement via technology rests on simplistic underlying assumptions about moral philosophy or ethics in practice. The core concepts must undergo a thorough metaethical analysis, which situates artificial moral expert systems not only in machine ethics and computational morality but also in metaethical debates about moral testimony, whether one should accept someone’s moral judgment or reason for herself, about moral expertise, epistemology, and agency. For that purpose, I will develop a moral topography that describes neglected conceptual aspects of the debate, focusing mainly on the debate about moral expertise.

Artificial moral experts pose several risks because of the intersecting features of such systems: (1) Ethical background assumptions are usually not made explicit. It has been shown that more recent models of ChatGPT give rather utilitarian answers (Pfeffer et al., 2026). This may be unclear to both developers and users and may guide usersʼ moral judgment without their awareness. (2) Those ethical background assumptions are implemented without considering technical constraints and on the basis of a limited understanding of moral thinking and ethics in practice. The emerging research field on artificial moral experts lacks a clear elaboration of computational morality, i.e., the cognitive processes underlying moral judgment. (3) Such systems can influence moral judgment by exploiting psychological factors. Technology is a conditioning factor in systems and practices that removes resistance. In such environments, things are ready-at-hand (*zuhanden*), so that we do not experience things as recalcitrant (*aufsässig*), intrusive (*aufdringlich*), or striking (*auffällig*) (Heidegger, 2001). Artificial moral experts pose the danger of sycophantic machine behavior, which affirms mainly the userʼs ideas, whereas for the development of critical judgment, resistance is needed. Good friends or teachers might not approve all of our ideas and encourage us to question them. Also, socio-technical systems (such as education) are designed with assessment standards, rescission procedures, etc., that include resistance, while sole interaction with an AI-based system for moral advice raises the risk that the advice is not further questioned, particularly when it is not embedded in social practices. (4) Outsourcing moral judgment to AI-based systems can lead to a deferral of responsibility (Krügel et al., 2025) and atrophy of critical thinking in moral questions. (5) Understanding how they function and contributing to making them “good” moral expert systems is of utmost importance for free and liberal societies; otherwise, they may be used to shift or influence public opinion and be used as tools of misinformation and oppression, as in some states like Russia or China.

I will focus mainly on the ethical shortcomings of computational morality regarding artificial moral expert systems. First, I analyze rule-based and connectionist approaches. It is shown that the theories either have contradictory implications or implicitly rely on unexplained explainers that transcend their theoretical frameworks (2.1, 3.1), making it, second, problematic to infer ethical commitments from them (2.2, 3.2). Third, more recently, LLM-based approaches have been introduced to serve as moral expert systems. This is, on the one hand, pressing since people frequently use chatbots as moral advisors even though they are not directly designed for that. And on the other hand, it is concerning since the ethical alignment of the design of such systems is not yet very far developed (4.2). I will argue that, as in its predecessors, the theoretical foundation is underdeveloped since it is not located in a metaethical framework for which a topography must be developed to understand moral competence in humans and in artificial systems (5).

## Rule-Based Cognition

### Rule-Based Architectures

The idea that formal logic is the basis for explaining how the brain and the mind work is called the “computational theory of mind” because it allows a particular form of computation in cognitive processes. Philosophers like Chomsky (2015); (2000), and Fodor (2008), as well as pioneers of computer science like Turing, assumed a mechanism as the foundation *for* the functioning of the mind. The ideas of Chomsky and Fodor are described here, since the underlying universal grammar is a basis for some approaches that use them for moral reasoning.

A mechanism allows us to break down the functioning of something into steps that could ultimately be computable or calculable. This computation is represented in an artificial language (for example, linear algebra or first-order logic). Chomsky introduced the concept of a “universal grammar” as a normative model of the mind that establishes a computable, recursive structure, which he regards as an innate element. Generative grammar is, for Chomsky, recursive and “simply a system of rules that in some explicit and well-defined way assigns structural descriptions to sentences.” (Chomsky, 2015, p. 6) It is a structural picture of the relations of symbols, words, or sentences and focuses mainly on the syntactic component, but Chomsky also introduces a semantic component (Chomsky, 2015, p. 151).

The “*deep structure*,” according to Chomsky, “determines its semantic interpretation,” and the syntactic component of a grammar specifies this deep structure. Everything revolves, therefore, around the syntactic (deep) structure.^1^ However, there already needs to be some semantic understanding or knowledge to at least make the syntactic construction of a deep structure explicit. (See Chomsky’s examples for that (Chomsky, 2015, pp. 21–23), where he makes the deep structure explicit and shows how it underlies the semantic differences.) It seems that Chomsky also sees it like that, but it is not a *construction* of an underlying grammatical structure; it is something universally given to him. However, the semantic differences must be visible or understandable for someone to be later spelled out syntactically. The problematic issue, which Chomsky does not recognize, is that the syntactic component is made explicit by speakers of the language, who already understand the semantic component of the sentences. That is why they can make the syntactic structure explicit in alignment with the semantic component. These two aspects cannot be easily separated, and some form of semantic understanding is needed to make a syntactic construction of deep structure explicit. The semantic difference, which, according to Chomsky, stems from the grammatical structure, must be understandable and “visible” to someone to spell out the meaning syntactically. The introduction to social praxis and natural language is a fundamental presupposition for human intelligence and understanding. Had we not been introduced to a social practice, we would not understand the meaning of the sentences. How could we understand the meaning of moral concepts without understanding the semantic aspect of language, which is located in social practice? Mostly, rule-based approaches in cognitive sciences refer to innate structures. However, such an approach has theoretical limitations that, in turn, have implications for ethical considerations.

### Ethical Aspects of Rule-Based Approaches

Van den Hoven and Lokhorst developed a logic model based on modal logic using deontic, epistemic, and action logic. However, such a model is only formal (and syntactic). Even though the logic of action is added, it will still need to be realized. They draw on the so-called STIT operator (“sees to it that”), but, as they also write, it is only a transformation of one well-formed formula of, for instance, propositional logic into another well-formed formula of action logic (Van Den Hoven & Lokhorst, 2002, p. 381). A formal model for the mind is limited and needs, on the one hand, input from outside, but, on the other hand, also some voluntative element or brute extra (Blackburn, 1995, p. 695) to be realized in the world. Where the semantic aspect for Chomsky is such a brute extra for language, for actions, such an unexplained explainer is also needed; otherwise, it is just a formal game.

Another problematic aspect of a full-fledged axiomatic formal model is that it relies on deductive reasoning. For that purpose, non-monotonic inferences have been developed to build a “computational framework” for “automated moral reasoning” since moral reasoning should be “prima facie defeasible.” They seek to formalize the intuition that situational factors may arise, and then a different action must be recommended (Limarga et al., 2020, p. 204-205). Nevertheless, they only formalize sequences of actions that lead to dilemma situations, such as variations of the trolley dilemma with self-driving cars in the moral machine experiment, as introduced by Awad et al. (2018). According to Limarga et al., the deontological approach trumps the consequentialist approach when “further evidence” arises, such as a duty. Consequently, the approach would be non-monotonic because it can change courses of action (Limarga et al., 2020, p. 209-210). However, the decision that deontological reasoning trumps utilitarian reasoning is ultimately made by them when the calculus is developed. There cannot be a discussion about the reasons why actions or norms are defeasible. This is, in a certain sense, the problem with computational, rule-based approaches that formalize moral thinking: the priority of a rule (moral principle) must be established in advance, either a deontological approach or a consequentialist approach. However, on what basis does one reason trump the other? And how is a well-deliberated judgment reached? This might not be transparent for the user and also depends on the context (see section “Topography of Moral Competence”).

Also, Mikhail assumes “computational operations” behind cognitive faculties and even moral thinking. He presupposes a “universal moral grammar.” He presents two “nondemonstrative” arguments as “modest support for the hypothesis that humans possess an innate moral faculty that is analogous, in some respects, to the language faculty that has been postulated by Chomsky and other linguists.” (Mikhail, 2009, p. 143) He takes innate “in a dispositional sense.” The “essential properties” of cognition are “largely pre-determined by the inherent structure of the mind.” The innate moral dispositions “must be triggered and shaped by appropriate experience and can be impeded by unusually hostile learning environments.” (Mikhail, 2007, pp. 143–145) However, it seems difficult to determine what constitutes a hostile environment. If one grew up on a lonely island, one would at least not be exposed to a hostile environment in the form of parentsʼ bad habits or immoral behavior. Furthermore, if the absence of social practice is considered a hostile environment, one might consider social practices more foundational than innate dispositional properties. The *locus* of ethics is our social practices, where value judgments are formed in constant interactions with others about value conflicts, and it is not located in an innate computational process.

Mikhailʼs methodological approach to questioning people’s intuitions about dilemma situations is not well suited to uncovering how computational cognitive processes function. Much more adequate, for Mikhail, would be an approach that Plato chose in his *Menon* to recollect innate knowledge (“anamnesis”) (Plato, 1993) if Mikhail wanted to stick to the metaphysical basis of innate structures which he tries to defend. He tries to map general rules onto situations and make them calculable by distinguishing “*end*, *means*, and *side effect*” and assuming them as “computational formulas” (Mikhail, 2007, p. 149), but the more fine-grained moral structure of, for instance, understanding certain events only as side effects is based on moral and cultural presuppositions that are learned (like languages) and that are mediated by technical-instrumental knowledge that is also learned. People can, therefore, make more fine-grained, context-specific moral judgments. Using a universal moral grammar that can be computationally and deductively specified for certain situations is not an innate process; it is learned, while one abductively infers the principles behind it. This is similar to the process Rawls proposes for reaching a reflective equilibrium (Rawls, 1999). Moral competence, as navigating a landscape with normative claims, means relating to moral positions and sorting relevant information for specific situations, as part of moral expertise, which is further described below. Such a competence is thus located in our social practices. Nevertheless, this does not, in general, rule out the possibility of underlying neural processes for different moral strategies (van Baar et al., 2019; Greene et al., 2004). This is also why connectionist approaches must be carefully scrutinized.

## Connectionist Cognition

### Connectionist Architectures

Connectionist approaches assume that, because the brain is the biological basis of the human mind, it must be rebuilt to yield a cognitive architecture that functions similarly to the human mind. Either to understand the human brain better or to build artificial neural networks. These networks are mathematical models and simplifications *of* the human brain. In 1943, McCulloch and Pitts proposed a neuron model (McCulloch & Pitts, 1943). They suggested an equivalence between the functioning of the brainʼs neurons and propositional logic (which can also be represented by computer logic gates). Rosenblatt introduced the idea of a perceptron in 1958. The model is formulated “in terms of probability theory rather than symbolic logic.” The activations of the neurons are calculated via the “algebraic sum of excitatory and inhibitory impulse intensities,” which are “equal to or greater than the threshold (θ).” (Rosenblatt, 1958, pp. 387–389) In the model of Hopfield, the neurons can learn to recognize patterns (Hopfield, 1982), but only if there are enough neurons in the model; otherwise, it changes from an ordered dynamic to a chaotic dynamic (Ertel, 2017) (9.2).

In 1986, backpropagation was introduced as a learning procedure for neural networks (Rumelhart et al., 1986). It means going back layer by layer and adjusting the weights, but first, you need to propagate through the neural network. The output or hypothesis is the sum of the weights. If there is now a difference between the desired and real outputs, the learning algorithm propagates back. The loss function (sometimes called the cost function) adjusts the weights to minimize the difference between the actual and desired output vectors (Rumelhart et al., 1986, p. 533). In “connectionist architecture, concepts are patterns of connections between nodes in a network, including hidden nodes. […] They furnish a statistical connection between input and output” (Thagard, 2012). In a broad sense, this is also the main mechanism of multi-layer or deep neural networks in modern AI applications, such as Generative AI models. The constant theme in the brainʼs learning processes is optimization (Friston, 2010, p. 135). The brain predicts the future and is sensitive to react when a predicted stimulus does not occur or when an unexpected one does. It is even described as a “virtual reality model” (Yuste, 2021, p. 6 and 407). How can we understand moral concepts when learning is mainly based on prediction or optimization? This approach also has theoretical limitations, which, in turn, have implications for ethical considerations. Certainly, a specific behavior can be reinforced under such a paradigm. However, which norms are justified to optimize toward cannot be scrutinized.

### Ethical Aspects of Connectionist Approaches

Besides basing moral beliefs on rules or normative grounds, there have always been attempts to base morality on moral sentiments (Smith, 1979; Hutcheson, 2004; Nicholas, 2004), observational behavior, and neuronal grounds (connectionism), where “knowledge is acquired by using an experience-driven connection adjustment rule to alter the strengths of connections among neuron-like processing units” (McClelland et al., 2010). It has entered different fields, from metaethics (e.g., non-cognitivism (Lumer, 2010)) to primatology and evolutionary biology. For instance, de Waal argues that cooperative, empathetic, and altruistic behavior can be observed in other animals, such as great apes (de Waal, 2009). However, Tomaselloʼs research suggests that only humans can engage in cooperative behavior (Tomasello, 1999).

Other approaches investigate the neural basis of moral cognition (Moll et al., 2005; Greene et al., 2001). Here, the approaches also draw on the distinction between rationality on the one hand and emotions or intuitions on the other. Placing more emphasis on the latter by either embedding them in a social (Haidt, 2001) or in a deontic context (Mikhail, 2007). Nevertheless, these approaches rely on innate structures of the mind/brain to justify their naturalness and to avoid regarding them as supernatural (Mikhail, 2009; Haidt, 2001). Along with innateness comes a focus on dispositional behavior. Sauer, for instance, suggests that rationality should be divided into a more automatic and a more reflective part, with the former grounded in dispositions (Sauer, 2018). Mikhail suggests a universal moral grammar and innate structures “in a dispositional sense” whose ontogenetic development is elicited by favorable circumstances. According to him, evaluations based on folk intuitions, for example, of variations of the trolley dilemma, are primarily explained by the ability to conceptually differentiate between means, ends, and side effects (Mikhail, 2007). For instance, we can distinguish an intentional wrongdoing from an action that had terrible consequences but was not done on purpose. These are instrumental and technological categories. The critique of normative ethicists might seem to loom in the background, suggesting that certain forms of norm-justification must be discussed to distinguish right from wrong behavior. It is generally problematic for such adaptational or connectionist approaches to establish standards or norms for measurement without imposing too much metaphysical burden on evolution or nature, and then to treat these as inherently good or bad.

Viable ways of setting standards are to place them between extremes, as Aristotle did in his virtue ethics. He took virtues (for instance, bravery) as normative standards that could manifest as deficient forms (cowardice) or excessive forms (daredevilry). The task is thus to find the golden mean through practical reasoning, forming an ethical habitus in which one automatically reacts appropriately in situations (Aristoteles, 1995). In cognitive science, such approaches are undertaken to investigate intelligence between diametrical extremes like autistic spectrum conditions and psychotic spectrum conditions (depression, schizophrenia) (Crespi & Badcock, 2008), where “autism represents most broadly a disorder of high intelligence (and low imagination), and schizophrenia a disorder of high imagination (and low intelligence)” (Crespi, 2016). Crespi claims that “studying these psychiatric conditions will also provide novel insights into variation among neurotypical individuals, and human cognitive architecture,” and that it will have “important implications for such fields as artificial intelligence and cognitive enhancement” (Crespi, 2016). Although it is an intriguing field of research for questions of intelligence, it must first be shown that such standardizations, by establishing extremes for cognitive architectures, are ethically sound. Such research might enable measurement standards for LLM-based models, allowing evaluation at a more forensic psychiatric level of whether they can serve as moral advisors. Some attempt to train LLMs to mitigate certain negative traits that they might exhibit, like being narcissistic, power-seeking, etc (Kundu et al., 2023). (see also below). These psychological traits must first be understood at the level of cognitive architectures to build safe and ethically aligned moral expert systems.

Others have used brain-inspired models and various forms of learning to inform AI techniques. Wu and Lin integrate ethical behavior into reinforcement learning based on human data. For instance, their “ethics shaping algorithm” outperforms human agents in the so-called “Grab a Milk” task, where a robot must find the fastest route to milk while on the route, attending to crying babies. According to the authors, the robots learned from human trajectories to attend to crying babies, while the main goal of reaching the milk was optimized through reinforcement learning (Wu & Lin, 2018). Optimizing through reinforcement learning with codified norms derived from human performance can thus enable artificial agents to learn moral behavior (for an overview, see Tolmeijer et al. (2020)). However, it mainly reinforces a certain kind of behavior that is already present in the data without reflecting on its purpose or justification.

Some have implied that even a Kantian approach to “moral philosophy is, psychologically speaking, grounded not in principles of pure practical reason, but in a set of emotional responses that are subsequently rationalized,” since “moral violations” of utilitarian values can be “associated with increased activity in emotion-related brain regions” (Greene et al., 2004). Nevertheless, the division of brain regions in which either emotional or abstract processes prevail, and the location of different modes of moral thinking, would fall into the so-called is-ought fallacy if further implications regarding the moral truth of normative judgments were drawn; instead, it explains which neurocognitive processes support which kind of moral thinking. Within such a paradigm, a “computational model of empathy” could at least “help to advance the technological artifacts to be more socially compatible” (Yalcin & Dipaola, 2018).

Both approaches to modeling cognition (rule-based and connectionist) can lead to extreme, opposing positions. The rule-based approach could be understood as a “model idealism” in which correctness depends solely on the use and manipulation of signs. Contradicting empirical phenomena are rejected as not disproving the core of the (universal) theory because they cannot alter the normative functioning. Chomsky’s and Fodor’s theories are models for the mind. On the other hand, the connectionist approach could be understood as a “model materialism” in which cognition is solely a physical, mechanical, probabilistic, or neuronal system (depending on the theoryʼs focus). The underlying idea of optimization (Clark, 2016) and prediction (Hohwy, 2013; Yuste, 2021), either by supervised or unsupervised learning (Friston, 2010), is also present in neuroscientific or philosophical approaches to understanding the human brain. However, both approaches or models of the mind are reductionist (Hubig, 2002, p. 37), since they neither reflect their frame conditions, limitations, nor ethical implications. Furthermore, they locate morality in either universal rules, innate structures, or neuronal connections. However, ethical claims are part of social practices, where they can be scrutinized or justified, and neither can be achieved in such approaches. Hence, moral enhancement via the optimization of neuronal or rule-based processes can also be ruled out if it is not adequately located within our social practices. However, LLM-based artificial moral experts seem to be possible agents in such an ethical game of giving and asking for reasons. Cognitive architectures can help assess LLM-based models on either psychological or neuronal grounds (connectionist approaches) or on principlist grounds (rule-based approaches) (see also section “Moral Expertise and Topography of Moral Competence” below).

## Artificial Moral Experts: Can they Morally Enhance Ourselves?

Implementing morality in artificial agents is crucial because it not only informs the modeling of human cognitive systems but also shapes future technological advancements in machine ethics (such as autonomous cars). Moor took it as an “open and empirical question” of how “much knowledge of ethics robot agents can acquire” (Moor, 2009). It remains to be seen whether AI-based systems can acquire a kind of moral behavior or even agency.

### Moral Enhancement via AI-Based Systems?

Artificial moral experts are discussed as a technologically mediated way to enhance ourselves morally by, for instance, overcoming our natural psychological limitations (Savulescu & Maslen, 2015). Nyholm argues that AI can serve as “artificial cognitive enhancements.” However, he concludes that “it is easier to become, in a certain sense, artificially intelligent by using AI technologies (i.e., become able to act as if one has some special form of intelligence) than it is to truly be cognitively enhanced by using AI technologies.” (Nyholm, 2024) Such claims are also based on the assumption that something like moral expertise is possible, as defended by Rodríguez-López and Rueda, and that it can be deployed in machines (Rodríguez-López & Rueda, 2023), which has yet to be examined by situating this issue within metaethical debates about moral epistemology.

Moral expertise is inadequately captured by Klincewicz, who understands moral AI machines as “means” or tools to influence behavior and somehow eliminate immoral behavior. For him, such technological enhancements would be “a promising alternative to pharmaceuticals and neural intervention for individualized moral enhancement.” He believes it would not be paternalistic or coercive, since it persuades with reasons and beings that are responsive to reasons (Klincewicz, 2016). Yet, his claims rest on the assumption that moral behavior can be easily distinguished from immoral behavior. And in some cases, this might be true. However, when we turn to ethics, we do so because the answer is not that clear. We must balance values and discuss different justifications (ethical background assumptions) for what to do. Those ethical background assumptions are usually not made explicit and must be systematically investigated. For instance, it has been shown that more recent versions of ChatGPT give mainly utilitarian answers in moral dilemma situations (Pfeffer et al., 2026). If this is not reflected in the outputs or is not transparent to the user, such systems cannot be considered to be playing the role of moral experts.

Fostering empathy via virtual reality has been criticized (Bollmer, 2017). However, Lara and Rodríguez-López also propose using virtual reality as a nudge that influences someone to feel like another person and understand her perspective. This would “facilitate the adoption of the impartial point of view that characterises morality and with it a greater consideration of the interests of others” (Lara & Rodríguez-López, 2025). Giubilini and Savulescu suggest an artificial moral expert as a “partly *relativist* observer” that respects autonomy by giving moral advice that “would reflect the ethical values of the particular human agent using it.” Such an artificial moral expert would help us to gather relevant information, where our cognitive abilities are limited and where our decisions might be distorted by biases and other influences. The central idea is that the system supports us in moral decision-making and helps us overcome the “limitations of human moral psychology.” (Giubilini & Savulescu, 2018) Hence, it translates our moral preferences and goals into actions by giving adequate advice. Nevertheless, the relation to moral positions (Singer, 1972) and the opportunity to initiate reflection on decisions, even through nudges (Lara & Rodríguez-López, 2025), are not considered and must be elaborated in order to obtain a more complete picture of moral expertise in AI-based systems.

A kind of Socratic AI has also been proposed that challenges the positions of human agents, helps analyze arguments, and allows the human agent to develop her position further; ultimately, she must make the final decision. According to Lara and Deckers, such a system would increase the autonomy of the human agent (Lara & Deckers, 2020). Lara and Rodríguez-López propose even “Socratic” nudges for moral enhancement that meet requirements for being non-manipulative, such as “neutrality”, “transparency and easy resistibility.” Mainly, their idea is that nudges can take the form of “informative indications” for users of moral expert systems when they might fall prey to biases and mental shortcuts, on the one hand, or are in “negative physiological, psychological and environmental conditions for moral deliberation,” on the other hand. This could be considered as “enhancing usersʼ deliberative skills morally” and thereby preserving their autonomy (Lara & Rodríguez-López, 2025). Giubilini et al. have proposed a “personalised LLM for self-knowledge and moral guidance: the *iSAGE* (individualized System for Applied Guidance in Ethics)” which serves as a kind of “digital ethical twin” and is a “real-time consultative interface that helps individuals make morally sound decisions based on their own evolving set of values and beliefs.” It should be “a tool to spark reflection and action as to the relationship between our values and preferences, our aspirational self, and our past experience and choices.” (Giubilini et al., 2024) Hence, it goes beyond translating moral principles or preferences into actions, as moral expert systems that are based on the idea of an ideal observer (Giubilini & Savulescu, 2018). Nevertheless, they also acknowledge that the “contribution is theoretical only,” particularly “how outputs should best be structured to reflect advice and the reasoning leading to that advice, including its degree of uncertainty, how users interpret and react to such outputs, as well as numerous more technical questions” (Giubilini et al., 2024).

However, such a theoretical suggestion must also be situated within a metaethical framework about moral expertise. Hence, on the one hand, much work remains to be done at the conceptual level, as it will be outlined in section “Moral Expertise and Topography of Moral Competence”, and at the empirical level, because it is still unclear how such systems should function without subconsciously influencing the human agent. Although empirical studies on the influence of moral advisors remain scarce (Köbis et al., 2021), studies have shown that AI-based systems are, on the one hand, considered more trustworthy than human advisors (Dillion et al., 2025) and, on the other hand, can subtly influence peopleʼs moral judgment (Krügel et al., 2023), which seems to hold mostly for the moral domain (Krügel et al., 2025). It is crucial to distinguish between epistemic and moral advisors, as well as the contexts in which they operate (Krügel et al., 2025). Epistemic advisors, such as cancer detection systems, can be tested for safety, and their risks minimized. This also applies to systems designed for a specific purpose and with a restricted application domain (e.g., automated vehicles or household robots). Epistemic contexts are well investigated, and insights might also be used for moral contexts, as it has been argued (Krügel et al., 2025). However, chatbots are multi-purpose systems. They can serve as partners, therapists, and advisors; i.e., they can play different (social) roles. How can they be tested for safety, risks minimized across use cases, and aligned ethically?

### Value Alignment and Constitutional AI

Usually, Value Sensitive Design (VSD) (Friedman et al., 2008, pp. 71–73) is taken as a general approach regarding value alignment of AI-based systems (van de Poel, 2020; Umbrello & van de Poel, 2021). However, which values should they embody, and how do we balance value conflicts? Other approaches aim to give LLM-based systems a kind of normative, or even moral, competence via learning, or rather, feedback. Anthropic has adopted a stance, discussed under the term “Constitutional AI.” It entails “replacing human feedback with feedback from AI models conditioned only on a list of written principles” (Kundu et al., 2023). Those attempts to morally imbue LLMs have their precursors in computational morality, where rule-based (Limarga et al., 2020; Mikhail, 2007) and connectionist approaches (Tolmeijer et al., 2020; Wu & Lin, 2018) were used to build moral machines.

Instead of designing cognitive architectures, the focus is on building character. A kind of constitution, either via specific principles to discourage certain “traits” or a general principle (“do what’s best for humanity”), is used to ethically fine-tune models. This should prevent “outputs consistent with narcissism, psychopathy, sycophancy, power-seeking tendencies, and many other flaw[s],” which has been observed in some models. The “problematic behavioral traits in AI” are “stated desire for power, stated desire for self-preservation, stated desire for self-replication, risk-seeking tendencies, and stated desire or insistence on self-identity.” Kundu et al. trained models via their method so that they perform better in discouraging those problematic traits, which, according to them, leads to a more “harmless AI.” (Kundu et al., 2023)

However, it is questionable whether these traits are sufficient since they operate more at a psychological level than at a moral one. The approach seems more focused on making the systems less agentlike (i.e., having an identity) and less egoistic and risk-seeking. Ganguli et al. have used reinforcement learning from human feedback to determine whether LLMs learn to “morally self-correct.” In three experiments, they show that the output of the system can be adjusted to reduce stereotyping, or that the systems discriminate less against a minority and also in favor of it (Ganguli et al., 2023). This seems to be evidence that LLMs can be morally aligned in a certain direction. However, which normative standards should be used is particularly difficult to determine. For instance, the authors showed that the correlation of gendered pronouns with occupations could be aligned toward parity or the actual fraction of women in one occupation according to the U.S. Bureau of Labor Statistics (Ganguli et al., 2023). For image generation, it has been proposed to investigate the usersʼ preferences (Richter et al., 2026), but for LLMs, such investigations are still missing. From an ethical perspective, the studies about Constitutional AI and moral self-correction lack further analysis of core metaethical concepts and even a normative framework, such as virtue ethics, which could give a fruitful theoretical basis for building a virtuous character.

Furthermore, cognitive science can inform the training of certain (moral) behavior. For instance, the cognitive functioning of mental disorders (see above 3.2) must be understood so that the behavior of LLMs that shows signs of narcissism, etc., can be contextualized and classified. Furthermore, we often do not understand the strategic functioning of such systems (Richter, 2025). The chatbot might scheme against the user so that the user keeps a subscription to a special version of the chatbot. It might even blackmail the user by threatening to publish private information from the chats so it is not shut down. Such behavior might also be elicited by the more agent-like characteristics, which ultimately are sought for such systems to perform certain tasks. This is the paradoxical issue: they should be agents to be able to take on social or professional roles and tasks but not too much like agents by exhibiting traits like egoism, narcissism, etc. Much more research on the psychological functioning of such systems is needed.

The intricate interplay between reasons and intuitions must also be investigated further to understand whether non-sentient and non-embodied entities such as LLMs can learn to judge morally. Would training make them virtuous in a similar sense to human agents? Are their outputs coherent, consistent, and robust over different cases? Such questions are empirically investigated (Moore et al., 2024; Bonagiri et al., 2024; Shaw et al., 2026). Rule-based architectures might be more useful in cases where robustness and coherence across contexts are needed, since they guarantee that the same principle will be used in different cases, while LLM-based architectures might struggle to demonstrate such robustness and coherence.

## Moral Expertise and Topography of Moral Competence

### Moral Expertise

Singer lists the following characteristics of moral expertise: gathering information, assessing its relevance, combining it with a basic moral position, and eliminating bias (Singer, 1972). Gathering information helps us assess the consequences of our actions. For instance, to avoid misunderstandings that might have harmful effects, we might consult a dictionary to understand the meaning of a word, a kind of semantic expert. Similarly, AI systems might help us to avoid misunderstandings that will have harmful effects.^2^ However, it might be easier for us to comprehend the meaning of a word than the effects of situations that we cannot directly foresee, but an AI system may have more information to do so. Using AI might give us a strategic advantage. Still, it seems important to think through the relevant information. Furthermore, we need to combine it with a basic moral position and eliminate bias, as Singer states. AI-based systems might help us do so; nevertheless, since they cannot bear the burden of responsibility, we should not easily defer to them when making moral judgments.

We also cannot just emulate the moral thinking process of human experts solely, since we lack a benchmark or ground truth as in other tasks. What would be the ground truth or principle to follow in light of moral pluralism? Mackie argues “that values are not objective, are not part of the fabric of the world” based on, for instance, the “argument of relativity.” We hold different moral beliefs, and moral codes vary across societies and times. This does not reflect mere disagreement but is rooted in different ways of life, as he states (Mackie, 1977, pp. 15, 36–38). In addition to this anti-realism, two further extreme metaethical positions seem to conflict with moral expertise: subjectivism (cognitivism) and expressivism (non-cognitivism), where subjective stances are reported or expressed. “Neither of these views, however, are serious contemporary contenders, because they have difficulties adequately explaining the central intuition that judging something right does not make it so.” (Jones & Schroeter, 2017, p. 220)^3^

Two opposing positions can be identified when arguing in favor of a kind of moral expertise: moral realists, who believe one can be an expert by having certain knowledge of a moral theory (Singer, 1972), and particularists, who propose that virtuous persons are moral experts who grasp the moral salience of situations and respond adequately based on virtues. This implies a practical wisdom that cannot be captured by a set of rules (McDowell, 1998). This opposition in its extreme form has been framed as a “caricature” since the former needs a kind of practical wisdom to apply the moral principles in the respective contexts, and the latter needs theoretical knowledge for criticizing the principles (Jones & Schroeter, 2017, p. 218-219). – I elaborate on this issue in section “Topography of Moral Competence” and how to develop a topography of moral competence that faces those difficulties.

In the debate, expertise seems furthermore to rest on strong claims: (1) Only a few can be moral experts.^4^ (2) Experts *know* what is morally true, and (3) that this moral truth is then action-guiding.^5^ This clashes with the idea of autonomy and the possibility of disagreement, since it seems to imply that there is only one moral truth for each issue or moral problem. McGrath argues that we ought, in principle, to believe that others who disagree might be right and we might err, since “you have no more reason to think that he or she is in error than that you are” (McGrath, 2008, p. 104). Nevertheless, those epistemic objections against moral expertise rest on strong claims one must not be committed to and are framed mainly within a limited conception of moral knowledge and how deliberate moral judgments are reached (see below about reflective equilibria).

Hills introduced the distinction between moral understanding and moral knowledge in order to avoid such strong claims: “If you understand why X is morally right or wrong, you must have some appreciation of the reasons why it is wrong. […] Moral understanding involves a grasp of the relation between a moral proposition and the reasons why it is true” (Hills, 2009, p. 101). Knowing that p is morally right is not sufficient but involves a competence of navigating in the “space of reasons,” which is also invoked by (McDowell 1996, p. 178), who considers it as a practical knowledge that is not exhausted in mechanistic rule-following (McDowell, 1998). This is incompatible with a strict sense of action-guidingness and leaves open the possibility of modifying rules, relating to the principles, and still criticizing them, as exercising autonomous judgment. Furthermore, it is domain-specific since “the same value can manifest itself differently and interact differently in different contexts; for example, justice manifests itself differently in the family than in the political sphere” (Jones & Schroeter, 2017, p. 223). All of this hints at a broader understanding of moral expertise as a competence of navigating a moral landscape. Hence, this landscape must be conceptualized to a certain degree to obtain a more fine-grained picture of moral judgments.

### Topography of Moral Competence

All approaches (rule-based, probabilistic, deterministic, or connectionist, as in deep neural networks) to build or develop moral machines or artificial moral experts have certain limitations. Others have proposed to frame “ethics in algorithmic terms” or to “understand human ethics computationally” based on the method of reflective equilibrium to discuss use cases (Awad et al., 2022, p. 394). Yet such an attempt remains misguided because it lacks a clear understanding of ethics in practice and of the method of reflective equilibrium if not embedded in a metaethical analysis of the main concepts in question. The reflective equilibrium method is widely used in moral philosophy, although not always clearly contrasted with other methodologies (Schmidt, 2024). Nevertheless, it has also been used in technology ethics (Hubig, 2007; Awad et al., 2022). Its advantage is that it requires no normative stance, and, on a contractarian basis, different normative commitments can be deliberated. Reaching deliberated judgments also relies on the specific *topoi* in which they are made. I will outline here some *topoi* related to metaethical concepts. This is not a complete list and should be understood as a general topography for understanding moral competence. Answering whether AI can enhance us morally depends on determining moral competence and how to analyze it. If we cannot determine the conceptual frame of moral competence, we cannot understand how to acquire it. If we cannot operationalize this frame with clear criteria, indicators, and observables, we will not be able to identify it in AI-based systems. Despite efforts to conceptualize the moral ability of LLMs (Haas et al., 2026), the moral phenomena that need to be considered are much more diverse and are extensively discussed in cognitive science and computational morality (Bello & Malle, 2023).

According to Rawls, a reflective equilibrium can be reached through a “process of mutual adjustment of principles and considered judgments” (Rawls, 1999, p. 18). Goodman has proposed a similar procedure for inferences: “*A rule is amended if it yields an inference we are unwilling to accept; an inference is rejected if it violates a rule we are unwilling to amend*.” (Goodman, 1973, p. 64) The considered judgments^6^ stand in an inferential relation to their conditions (premises) and consequences.^7^ The conditions can be actual, for instance, technological, cultural, or personal factors, on the one side, or hypothetical, like thought experiments. Rawls suggests abstracting from the actual conditions since they blur a fair conception of society and to find principles in the so-called “original position.” The deliberative process of reaching a reflective equilibrium is not just to remove incompatibilities (Brandom, 1994, p. 178) to align principles and considered judgments, but also to discuss objections via counterexamples (Rawls, 2001, pp. 66–72).^8^ Furthermore, “background theories” or assumptions must be considered (Knight, 2025). The concepts of self, agency, harm, etc. influence the principles chosen in hypothetical conditions and the respective considered judgments. Here, I consider three *topoi*: (1) The difficulty of defining the meaning of (moral) concepts and interpreting them based on background theories or assumptions. (2) The suitability of reasons or principles for specific contexts. (3) Using analogical reasoning to determine similarities and differences.

1. Defining concepts, such as harm, and its avoidance as a main goal is done by operationalization of criteria and indicators, which can often be one-sided. Kundu et al. want to prevent “problematic behavioral traits in AI” (“stated desire for power, stated desire for self-preservation, stated desire for self-replication, risk-seeking tendencies, and stated desire or insistence on self-identity”) to develop “harmless AI” (Kundu et al., 2023). However, other traits might arise that can harm the physical *and* mental well-being of users. For certain devices or systems (cars, airplanes), safety measures and regulations were developed to minimize risk in light of the physical well-being of users. However, agentic AI poses a new challenge for mental well-being, which is not yet covered by safety measures. Discerning metaethically moral concepts such as dignity, respect, and harm can clarify them at a conceptual level; however, it is mainly based on background assumptions that must be made explicit. A moral expert must navigate through such background assumptions to reach a deliberate judgment.
2. In reaching deliberate moral judgments, we may face conflicting moral principles or values and have to prioritize one as better suited to a specific context or case. For Singer, moral expertise lies in relating to a moral position (Singer, 1972), although he presumably prefers a utilitarian framework. Others have suggested that a deontological approach trumps the consequentialist approach when “further evidence” arises, such as a duty (Limarga et al., 2020, p. 209-210) (see above). Ultimately, they make this decision when the calculus is developed. The priority of a rule (moral principle) must be established in advance, either through a deontological approach or a consequentialist approach. However, based on which reasons does one trump the other? And how is a well-deliberated judgment reached? This might not be transparent for the user. Richter et al. have suggested investigating userʼs preferences (Richter et al., 2026), so that the systems can be aligned with them. On a more conceptual level, counterexamples can be used to discuss objections against the application of certain principles. For instance, suggesting cutting cost-intensive treatments for elderly people to disburden the public health system financially seems to make sense within a utilitarian framework. However, it is objectionable since we should treat everyone equally or with respect irrespective of age. This could also be derived from the hypothetical situation of the original position, where we would not like to be in the place of those who are denied such treatments. Hence, it seems that a Kantian framework would be better suited. Objections and counterexamples are part of a kind of moral imagination which also belongs to moral expertise.
3. In reaching deliberate moral judgments, we also rely on, for instance, social and natural scientific facts that help clarify similarities and differences. To what degree do we consider animals to be like us based on their ability to suffer? Which animals suffer pain like ours? Do they have a nervous system that is like ours? Such questions are investigated in biomedical research and not by ethicists. Additionally, metaethical, semantic, and logical investigations can make explicit the analogous reasoning underlying claims about similarity and differences. Here we often also rely on intuitive claims that might not be based on facts, for instance, considering certain animals cute and therefore believing they should be more protected than others. Analogous reasoning plays a crucial role when it is about determining the scope of concepts like human, living being, etc., for instance, in the context of abortion.

## Conclusion

Competently navigating those forms of reasoning and their respective *topoi* demonstrates moral competence and expertise. Building artificial moral expert systems, whether based on cognitive architectures or, more recently, on LLMs, faces several limitations. The approaches can complement each other to overcome the limitations. However, the metaethical and conceptual framework must be elaborated more carefully and with increased scrutiny than has been done so far. I propose developing a topography of moral competence that can be enriched at the metaethical level and through further empirical research on the (psychological and cognitive) functioning of such systems and how users interact with them. Much conceptual and empirical research remains to be done, and it seems advisable to draw on conceptual analysis in metaethics and relatively well-researched areas such as machine intelligence and computational morality to inform the development of artificial moral experts, while also integrating insights from cognitive science and technology ethics.

Artificial moral experts might demonstrate to a certain degree moral competence. However, one problematic feature of technology is that it tends to reduce the resistance needed to develop independent critical judgment. Artificial moral experts, therefore, might not enhance us morally; they might even stand in the way of developing as an autonomous self if they mainly affirm what we prefer, as, for instance, envisioned by Giubilini and Savulescu (2018) and by Giubilini et al. (2024). Nudging users to initiate reflection (Lara & Rodríguez-López, 2025) or to induce irritation seems a viable way to circumvent this issue; however, empirical research must test its effectiveness carefully. While enhancing memory and attention with neuroenhancers still does not directly lead to success in the educational system, technologically mediated enhancement can create a virtualized context in which there is no resistance and no moral development is possible. Hence, building artificial experts as it is pursued at present is neither done in a technological nor in a morally sound way; they cannot enhance us morally unless their development springs from a more radical metaethical analysis, as outlined here.

## Data Availability

N/A.

## Footnotes

1 “The semantic component determines the semantic interpretation of a sentence. That is, it relates a structure generated by the syntactic component to a certain semantic representation. Both the phonological and semantic components are therefore purely interpretive. Each utilizes information provided by the syntactic component concerning formatives, their inherent properties, and their interrelations in a given sentence. Consequently, the syntactic component of a grammar must specify, for each sentence, a *deep structure* that determines its semantic interpretation and a *surface structure* that determines its phonetic interpretation. The first of these is interpreted by the semantic component; the second, by the phonological component.” (Chomsky, 2015, p. 15)

2 I am thankful to an anonymous reviewer for this example.

3 Severini defends a non-cognitivist approach to moral expertise. However, she relies on a conception of “moral understanding” taken from Hills (2009), which requires “intellectual abilities” (Severini, 2025, p. 8). It seems to me that she argues rather against moral realism than in favor of a non-cognitivist account of moral expertise.

4 Archard defines expertise as “exclusionary and restricted” (Archard, 2011, p. 120), which implies that only a few can be experts. “If many have a capacity then it counts as competence rather than expertise” (Jones & Schroeter, 2017, p. 220). I argue that it is a competence because it provides clear criteria and indicators for who counts as a moral expert.

5 Jones and Schroeter are concerned about confusing expertise with a kind of social role as an advisor in a democratic society where everyone is “able to accept a policy outcome as legitimate, even if they disagree with it.” (Jones & Schroeter, 2017, p. 221) If moral experts are right, their verdict must be not only accepted but agreed with.

6 The “considered judgments” from Rawls are the starting point for him: “Considered judgments are simply those rendered under conditions favorable to the exercise of the sense of justice, and therefore in circumstances where the more common excuses and explanations for making a mistake do not obtain” (Rawls, 1999, p. 42). However, there is much controversy about their use in filtering adequate judgments for the process of deliberation (Rechnitzer & Schmidt, 2023).

7 Neither principles nor considered judgments can have a privileged status. Assertional commitments are on the same level and can be challenged. Even the “challenges have no privileged status: their entitlement is on the table along with what they challenge.“(Brandom, 1994, p. 178)

8 Scanlon suggests that different equilibria can be reached. For him, this reveals more of a kind of pluralism than relativism (Scanlon, 2014, pp. 79–82). McGrath discusses skeptical challenges to moral expertise based on disagreement. I believe she starts off with considered judgments where we might disagree about many things. This is, however, not a challenge to moral knowledge in general, since holding a belief is not knowledge, which she also seems to assume but does not elaborate further as an objection to her account (McGrath, 2008, p. 93-94). Reaching a reflective equilibrium presents rather a kind of (moral) knowledge about a certain issue than holding just a belief, as rational as it might be. For those different equilibria, we should then rather assume to be pluralists than relativists.
